# Supporting and Non-Stigmatizing Communication in the Process of Weight Change: The Role of Motivational Interviewing

**DOI:** 10.3390/nu18081213

**Published:** 2026-04-11

**Authors:** Justyna Nowak, Anna Bartosiewicz

**Affiliations:** 1 Department of Metabolic Disease Prevention, Faculty of Public Health in Bytom, Medical University of Silesia, 41-900 Bytom, Poland; 2 University of Rzeszow, Collegium Medicum, Faculty of Health Sciences and Psychology, 35-959 Rzeszów, Poland; 3 University Center for Research and Development in Health Sciences, 35-310 Rzeszów, Poland

**Keywords:** obesity, motivational interviewing, behavior change, patient-centered communication, weight management, non-stigmatizing communication

## Abstract

Obesity is a complex public health issue requiring a holistic, interdisciplinary approach. The aim of this narrative review is to present motivational interviewing as a communication-based intervention that supports non-stigmatizing, patient-centered care for individuals with excess body weight, with particular emphasis on fostering intrinsic motivation and sustainable lifestyle change. This narrative review examines motivational interviewing (MI) as a tool to support non-stigmatizing, patient-centered communication and promote behavior change in adults, children, and adolescents with overweight or obesity. Evidence from original studies, systematic reviews, and randomized controlled trials suggests that MI can help improve healthy eating, increase physical activity, and strengthen confidence in maintaining lifestyle changes. Its effectiveness is linked to communication based on empathy, partnership, acceptance, and support, free from stigma, which enhances patient autonomy, and encourages active engagement. Despite promising results, research gaps remain regarding the long-term effectiveness of MI and its integration into routine clinical practice.

## 1. Introduction

Obesity is a complex and multifaceted public health issue. It is a chronic condition with a multifactorial etiology, involving biological, psychological, and social aspects, and therefore requires a holistic approach to medical care and interdisciplinary support. Effective diagnosis and treatment of obesity should integrate knowledge from medicine, psychology, dietetics, and public health, addressing both the physical and psychosocial needs of patients with obesity. Collaboration among specialists is essential for developing effective therapeutic strategies and providing long-term support for individuals with excess body weight [1,2,3].

When working with individuals with overweight and obesity, where the goal is to change lifestyle behaviors aimed at weight reduction, providing dietary recommendations, advice on physical activity, or general lifestyle guidance alone is often insufficient. An approach focused solely on identifying deficits and offering ready-made solutions rarely leads to lasting effects when the aim is to modify habits and achieve long-term behavior change. The effectiveness of weight reduction interventions is significantly increased by appropriate, supportive communication between the specialist and the person receiving care, as well as by interpersonal support provided both by professionals working with individuals with obesity and by the close social environment, including family members, peers, and other specialists involved in the healthcare system [4,5,6]. Healthcare professionals working with individuals with excess body weight should therefore combine clinical expertise with communication skills that emphasize respect, neutrality, and empathy, which supports both children and adults in the process of behavior change [7].

Weight stigma and discrimination are common experiences among individuals with excess body weight and are linked to a range of psychosocial, social, and health consequences. In adults, weight-based discrimination is among the most frequently reported forms of bias, while in adolescents it often appears as bullying or harassment in schools. Those who experience weight stigma are more likely to report lower self-esteem, social withdrawal, stress, and behaviors that contribute to weight gain, such as overeating or avoiding physical activity. Moreover, experiences of unsuccessful weight loss, weight regain, or concerns about body appearance can intensify negative emotions and make maintaining healthy habits more difficult. Recognizing and understanding these experiences is essential for designing healthcare and weight management interventions that are empathetic, non-stigmatizing, and tailored to individual patient needs, as well as for developing policies and support systems that foster inclusivity and well-being for people with excess body weight [8,9,10,11].

In this context, motivational interviewing (MI) can be an effective tool to support non-stigmatizing, patient-centered communication, while also enhancing intrinsic motivation and fostering collaborative relationships in the process of health behavior change [6,12,13,14].

The aim of this paper is to present the role of motivational interviewing as a communication-based intervention that supports non-stigmatizing, patient-centered care for individuals with excess body weight, with particular emphasis on its role in fostering intrinsic motivation and sustainable lifestyle change.

## 2. Materials and Methods

This narrative review was based on a search of scientific articles in the PubMed, Scopus, and Google Scholar databases. The search used combinations of keywords such as: “obesity” OR “overweight”, “motivational interviewing” OR “MI”, “health communication” OR “patient-centered communication”, “weight stigma” OR “weight discrimination”, and “behavior change” OR “lifestyle intervention”. Only articles published in English were considered. Although no strict publication period limits were applied, the analysis primarily focused on studies from the last 20 years to ensure inclusion of current research trends and recent evidence.

This review included original research articles, systematic and narrative reviews, as well as studies describing clinical and behavioral interventions using MI in both adults and children. The selection focused on foundational work on obesity and its biopsychosocial determinants, as well as key sources on the theory and practical application of MI. Studies on patient-centered communication, non-stigmatizing approaches, and behavior change strategies in the context of individuals with overweight or obesity were also considered.

The articles examined MI in a range of settings, including primary care, specialized behavioral or lifestyle programs, and family-based pediatric interventions. Both randomized controlled trials and meta-analyses were included to capture a broad picture of MI’s effectiveness in supporting weight management, promoting healthy behaviors, enhancing self-efficacy, and improving psychosocial outcomes. The review considered interventions delivered by a variety of professionals, including doctors, nurses, psychologists, dietitians, and multidisciplinary teams.

Studies were excluded if they were unrelated to obesity or MI, were opinion pieces or commentaries without empirical data, or lacked enough methodological detail to evaluate the intervention or its outcomes.

By combining evidence from both adult and pediatric populations, this review aimed to give a comprehensive look at how MI is used in obesity care, highlighting its potential benefits as well as the methodological limitations of the available research.

## 3. Discussion

### 3.1. Motivational Interviewing: Theoretical Foundations

The concept of MI comes from experiences in treating alcoholism and was first described by Miller in 1983. Later, Miller and Rollnick developed it into a coherent theory, defining MI as a client-centered counseling style aimed at supporting behavior change by helping individuals explore and resolve ambivalence. This method is particularly useful for people who are reluctant or uncertain about changing their behavior. Its main goal is to strengthen internal motivation so that change comes from the patient’s own initiative rather than being imposed externally. MI has been widely used in the treatment of conditions partly related to behavior. It has been applied and studied in contexts such as alcohol misuse, drug addiction, smoking cessation, weight loss, adherence to medical advice and follow-up visits, increasing physical activity, and management of asthma and diabetes. Various healthcare professionals have used MI, including psychologists, physicians, nurses, and midwives [15,16,17].

MI is a collaborative, person-centered, and goal-oriented style of communication. Its aim is to strengthen a person’s own motivation and commitment to change. It is a method of helping people deal with the common problem of ambivalence toward change, with particular attention to change-related language. MI seeks to elicit and explore personal reasons for change in an atmosphere of acceptance and compassion, which supports the development of internal motivation and engagement in achieving a specific goal [4,5,6].

**Figure 1:**
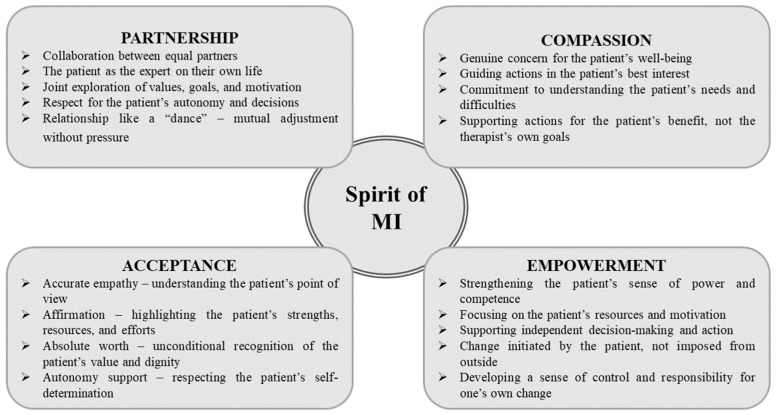
Core elements of the spirit of MI, illustrating the key principles that guide a collaborative, person-centered approach to supporting behavior change. Adapted by the authors based on [4,5].

During an MI session, the conversation may appear light, relaxed, and spontaneous. In reality, however, it follows a well-structured process based on specific principles and stages, where both the mental and emotional attitudes of the interviewer toward the patient are crucial [4,5,6]. An MI conversation is grounded in the spirit of MI, which encompasses Partnership (a collaborative relationship without an expert–patient hierarchy), Acceptance (recognizing the person’s dignity, demonstrating accurate empathy, supporting autonomy, and affirming strengths), Compassion (genuine concern for the person’s well-being and acting in their best interest), and Empowerment (highlighting the person’s own resources, motivation, and autonomy). The conversation is designed to elicit the person’s own reasons and ideas for change while avoiding criticism or negative attitudes [4,5]. The core elements of the spirit of MI, highlighting the key principles that guide a collaborative, person-centered approach to supporting behavior change are illustrated in Figure 1.

**Figure 2:**
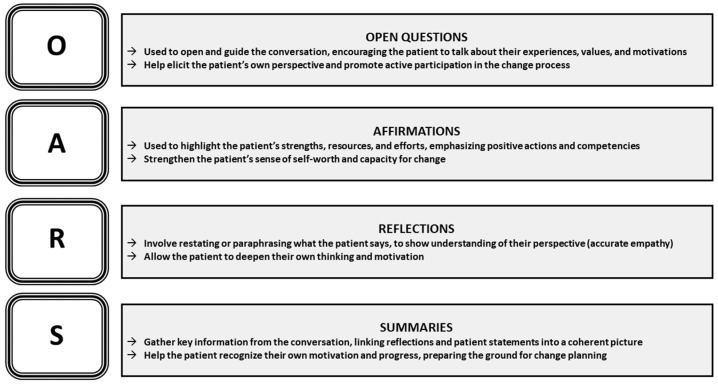
Core skills of MI (OARS). Adapted by the authors based on [4,5].

To support these processes, MI employs core skills (Figure 2), namely, Open Questions, Affirmations, Reflections, and Summaries (QARS), which facilitate active listening, reinforce patient autonomy, and help draw out the patient’s intrinsic motivation for change [4,5].

**Figure 3:**
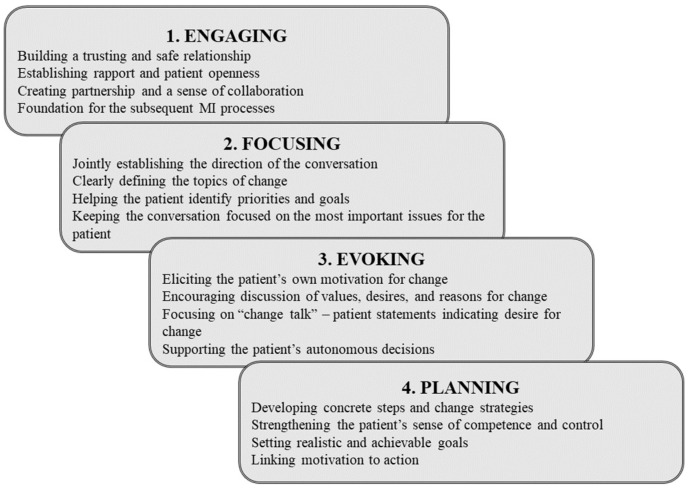
The four main processes of MI. Adapted by the authors based on [4,5].

In MI, the conversation is structured around four main processes: engaging, focusing, evoking, and planning (Figure 3). Engaging builds a trusting and empathetic relationship, creating a safe space for open communication. Focusing establishes a shared goal for the conversation and a clear direction for action. Evoking explores the patient’s internal motivation and personal reasons for change. In the final planning stage, the patient and interviewer develop specific, realistic steps to achieve lasting changes in health behaviors. Together, these processes support the patient in discovering their own motivation and making conscious, autonomous decisions throughout the change process.

MI is standardized and can be certified using the MITI (Motivational Interviewing Treatment Integrity) scale. This scale was developed as a systematic tool to assess the quality of motivational conversations. It allows identification of behaviors that are consistent or inconsistent with MI principles and evaluates how well a conversation meets the method’s standards [18,19,20,21,22].

### 3.2. Evidence of MI Across the Lifespan

MI is a well-known and scientifically studied counseling method, recognized as an effective intervention strategy for lifestyle-related problems and chronic diseases. Numerous randomized controlled trials and meta-analyses have shown that MI is more effective than traditional advice-giving in addressing various behavioral problems and health conditions. Over the past 15 years, the method has been widely adapted and tested in the context of different behaviors related to chronic diseases [15,16,23,24,25,26].

MI is a strongly evidence-based method. By 2022, more than 1900 controlled clinical trials had been reported, and over 200 systematic reviews and meta-analyses on MI had been published. Most studies (between two-thirds and three-quarters) showed significant benefits of using MI, confirming its effectiveness in supporting behavior change across various clinical and social contexts [17].

#### 3.2.1. Adults with Overweight and Obesity

Interest in MI as a method to support changes in health behaviors in people with excess body weight emerged several decades ago. This approach has been widely studied in the context of communication aimed at supporting patient motivation and autonomy [6,27]. Excess body weight increases the risk of developing non-communicable chronic diseases and premature mortality. In this context, achieving a lasting, long-term lifestyle change is a key goal in the treatment of obesity and the prevention of diseases related to excess body weight [28,29,30,31].

Despite these advantages, implementing lasting lifestyle changes is not simple, because adults with excess body weight often experience ambivalence toward changing their behaviors. This ambivalence is influenced by previous unsuccessful attempts at weight loss, stress, and weight-related stigma. Additionally, research on internalized weight stigma shows that these individuals may experience high levels of weight-related distress. In a broader social context, persistent obesity stigma is often overlooked, and blaming individuals for their excess weight makes it harder to create an empathetic and supportive environment, which is essential for effective communication-based interventions [32,33].

To address these challenges, MI is used to support changes in eating habits, increase physical activity, improve adherence to therapeutic recommendations, and strengthen long-term engagement in weight management. Through empathetic communication, collaboration, and respect for patient autonomy, MI creates a non-judgmental communication environment that encourages reflection on personal choices and informed decision-making. This approach supports lasting behavior change rather than just short-term weight loss [4,5,27,34,35].

In a systematic literature review including 24 randomized controlled trials (1994–2014) in adult populations, the effectiveness of MI in weight reduction was evaluated. MI interventions were usually delivered individually by various healthcare professionals and compared with standard care. Nine studies (37.5%) showed a statistically significant weight loss in the MI groups compared with control groups, while in thirteen studies (54.2%), participants achieved at least a 5% reduction in baseline body weight. These results suggest that MI has the potential to support patients in primary healthcare in achieving lasting weight loss by enhancing motivation and autonomy in the behavior change process [27].

A systematic review and meta-analysis of randomized controlled trials showed that MI can support weight loss in people with overweight and obesity. Participants who took part in MI sessions lost on average 1.47 kg more than those in control groups, and their BMI decreased by about 0.25 kg/m^2^. Although the effect is moderate, the results indicate that MI can be an effective tool for weight management. This method fits within a patient-centered approach, in which the professional and the patient work together as partners rather than in a traditional hierarchical relationship. MI also helps support individuals who are ambivalent about lifestyle changes and may be even more effective when combined with behavioral programs that promote weight loss [36].

The effectiveness of MI has been well established in meta-analyses, and recent randomized controlled trials (RCTs) further confirm its role in supporting weight loss in adults with overweight and obesity. In one RCT involving 217 women with overweight and type 2 diabetes, participants who received MI sessions as an adjunct to an 18-month group behavioral program achieved significantly greater weight loss at 6 and 18 months, with improvements in weight control linked to better adherence to the program; HbA1c reductions were significant only at 6 months, and benefits were smaller among African American women [37]. In a single-blind RCT of 100 women with overweight and obesity, participants receiving MI scored significantly higher on the Weight Efficacy Lifestyle scale and all its subscales compared with the control group, highlighting the effectiveness of MI in enhancing self-efficacy for weight management [38]. In a multicenter cluster-randomized RCT including 864 patients, those in the MI group who attended 32 group sessions achieved greater weight loss after 24 months than the control group (mean 2.5 kg vs. 1.0 kg; *p* = 0.02), and the proportion of participants losing ≥5% of body weight was higher in the MI group (26.9% vs. 18.1%; *p* = 0.04). The MI intervention also led to improvements in lipid profiles, including triglyceride levels and the APOB/APOA1 ratio [39].

Overall, both meta-analyses and randomized controlled trials consistently demonstrate that motivational interviewing is an effective, evidence-based approach for supporting long-term weight loss, improving self-efficacy, and promoting healthier lifestyle behaviors in adults with overweight and obesity.

#### 3.2.2. Children, Adolescents, and Family-Based Interventions

MI has also been explored as a strategy to support healthy behaviors in children and adolescents, often involving family-based approaches. These interventions aim to engage both young individuals and their caregivers in fostering sustainable lifestyle changes, including improved nutrition, increased physical activity, and weight management [40,41,42,43,44,45,46]. The 2023 American Academy of Pediatrics guidelines recommend the use of MI in the evaluation and management of pediatric obesity, emphasizing its role in engaging both patients and families. MI is particularly useful for assessing readiness to change, supporting behavioral action, and helping children and families regain momentum after setbacks, with the focus of counseling shifting from parents to the adolescent as the child gains autonomy [42].

Results of a meta-analysis of 25 studies show that parent-involved MI can improve diet, physical activity, oral health, screen time, and even body mass index (BMI) in children [43]. A systematic review and meta-analysis of 31 studies published between 2005 and 2022 showed that MI can lead to small but noticeable short-term benefits in health behavior change in children. These effects most often included lower calorie, snack, and fat intake, as well as higher levels of physical activity, while effects on other outcomes, such as screen time or dairy consumption, were limited [44]. A review of 26 randomized controlled trials shows that MI can meaningfully support children and adolescents with overweight and obesity. The studies reported not only improvements in body weight and health-related behaviors, but also better quality of life and reduced depressive symptoms. MI therefore appears to be a promising approach, especially when delivered as part of long-term, comprehensive, and family-based care provided by a multidisciplinary team [45]. A systematic literature review and meta-analysis of 33 studies evaluated the impact of MI on anthropometric parameters in children, such as BMI, waist circumference, and body fat percentage, indicating the effectiveness of MI in improving these measures [46].

In summary, research suggests that motivational interviewing can be an effective approach for helping children and adolescents with overweight and obesity. It may be associated with improvements in body measurements, promote healthier eating and increase physical activity, enhance confidence in making lifestyle changes, and support overall quality of life, particularly when delivered as part of long-term, family-based, and multidisciplinary programs.

### 3.3. Research Gaps and Future Directions

Despite growing evidence for the effectiveness of MI in adults and children with overweight and obesity, significant research gaps remain. The long-term effectiveness of MI in maintaining behavior changes and weight loss is not yet fully established. Some meta-analyses suggest that in adults with obesity, MI does not produce statistically significant effects on weight loss and BMI, which may be due to methodological limitations such as a small number of studies, poor reporting of intervention quality, or lack of control over protocol adherence [47]. Similarly, in adolescent populations, MI used alone has not shown a significant impact on the treatment of overweight and obesity, which may be related to small sample sizes, moderate risk of bias, and short follow-up periods [48].

Future research should examine the long-term effects of MI and how the quality and delivery of interventions influence their effectiveness, as well as investigate how MI can be integrated into routine clinical practice and multidisciplinary programs. This approach will improve understanding of how MI supports behavior change and weight management and facilitate the design of effective programs for adults, children, and adolescents. Additionally, future studies should more consistently report intervention fidelity using standardized tools, such as the Motivational Interviewing Treatment Integrity (MITI) scale, since MI requires specialized knowledge and practical training and is not easily replicable without proper preparation.

## 4. Conclusions

MI is a promising tool for supporting behavior change related to weight loss in adults and in children and adolescents with overweight and obesity. Evidence indicates that MI can promote healthier eating habits, increase physical activity, and strengthen individuals’ confidence in maintaining lifestyle changes over time. Its effectiveness is largely attributed to communication grounded in empathy, partnership, acceptance, and support, which is non-stigmatizing, non-judgmental, enhances patient autonomy, and facilitates active engagement in the change process. At the same time, significant research gaps remain regarding the long-term effectiveness of MI and its implementation in routine clinical practice, highlighting the need for further high-quality studies and well-designed implementation strategies.

## 5. Study Limitations

Despite the authors’ efforts, this work has several important limitations. Due to its narrative review, the review is selective and may be subject to potential bias in literature selection. Although high-quality studies were included, the review may not fully reflect the entire scope of new perspectives or unpublished data, given the large number of studies on MI across different contexts.

## Data Availability

Not applicable.

## Abbreviations

The following abbreviations are used in this manuscript:

MI	Motivational Interviewing
MITI	Motivational Interviewing Treatment Integrity
BMI	Body Mass Index
